# FT3 combined with the TyG index for risk stratification of mild cognitive impairment in euthyroid patients with type 2 diabetes

**DOI:** 10.3389/fendo.2026.1802217

**Published:** 2026-06-22

**Authors:** Yi Su, Lu Chen, Jing Huang, Haorui Lyu, Wei Feng, Jun Zhan, Yuyao Li, Zhanyi Ye, Xuan Liu, Changjiang Ying

**Affiliations:** Department of Endocrinology, The Affiliated Hospital of Xuzhou Medical University, Xuzhou, China

**Keywords:** FT3, mild cognitive impairment, risk stratification, TyG index, type 2 diabetes

## Abstract

**Introduction:**

Cognitive impairment is increasingly recognized as an important central nervous system complication of type 2 diabetes mellitus (T2DM). Thyroid hormones (THs) and insulin resistance are both implicated in brain metabolism; however, the associations of free triiodothyronine (FT3) and the triglyceride–glucose (TyG) index with cognitive impairment in euthyroid patients with T2DM remain incompletely understood.

**Methods:**

This retrospective cross-sectional study enrolled 525 euthyroid hospitalized patients with T2DM between September 2023 and March 2025. Cognitive function was assessed using the Montreal Cognitive Assessment (MoCA), and mild cognitive impairment (MCI) was defined as a MoCA score of 19–25. Logistic regression analyses were used to evaluate the associations of FT3 and the TyG index with MCI. Restricted cubic spline (RCS) models were applied to explore dose–response relationships and potential nonlinearity. Receiver operating characteristic (ROC) curve analyses were performed to assess the discriminative ability of FT3, the TyG index, and their combined model. The DeLong test was used to compare differences in AUCs between models. Calibration analysis and decision curve analysis (DCA) were further conducted to evaluate the predictive agreement and potential clinical utility of the combined FT3 - TyG model.

**Results:**

Lower FT3 levels were independently associated with a higher odds of MCI, even within the euthyroid range, exhibiting a significant linear negative dose–response relationship. The TyG index showed a nonlinear, asymmetric inverted J-shaped association with the odds of prevalent MCI, with a clear inflection point at TyG = 9.24, above which the estimated odds increased more rapidly. ROC analysis demonstrated good discriminative performance for FT3 alone (AUC = 0.761, 95% CI: 0.721–0.802), whereas the TyG index alone showed limited discriminative value (AUC = 0.571, 95% CI: 0.520–0.622). The combined FT3–TyG model yielded an AUC of 0.766 (95% CI: 0.726–0.807), which was not significantly higher than that of FT3 alone. However, within individuals with higher TyG levels, FT3 provided additional granularity for distinguishing those with a higher likelihood of MCI, supporting its role in risk stratification across heterogeneous metabolic profiles.

**Conclusion:**

In euthyroid patients with T2DM, physiological variation in FT3 remains independently associated with cognitive status, while the TyG index is nonlinearly associated with cognitive impairment, with an identifiable metabolic threshold. The combined FT3–TyG model demonstrated good discriminative ability, suggesting that these readily accessible, noninvasive metabolic indicators may be useful for identifying individuals with a higher likelihood of MCI and for cognitive risk stratification in T2DM.

## Graphical Abstract

**Figure:**
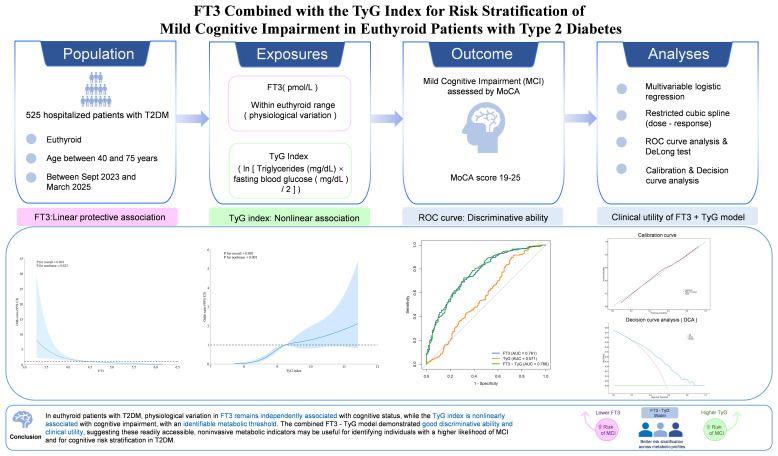

## 1 Introduction

Type 2 diabetes mellitus (T2DM) has become one of the most significant global public health challenges, characterized by persistent chronic hyperglycemia (1). Beyond traditional microvascular and macrovascular complications, cognitive impairment is increasingly recognized as an important central nervous system complication of T2DM (2). Epidemiological studies have shown that patients with T2DM have an approximately 50%–100% higher risk of developing mild cognitive impairment (MCI) compared with non-diabetic individuals (3). As a prodromal stage of dementia, early identification of MCI in patients with T2DM is of substantial clinical importance for delaying cognitive decline and improving long-term outcomes (4). At present, reliable biomarkers for early risk assessment of cognitive impairment in T2DM are still lacking, highlighting the urgent need to identify accessible metabolic indicators for early detection of high-risk individuals.

Thyroid hormones (THs) are essential regulators of brain development and play important roles in maintaining central nervous system homeostasis and adult neurobiological function (5). In older adults, thyroid dysfunction has been investigated as a potentially modifiable factor related to cognitive impairment and dementia, although existing evidence remains heterogeneous and partly inconsistent (6).Accumulating evidence has linked thyroid dysfunction to Alzheimer’s disease and dementia (7), and a recent systematic review and meta-analysis showed that overt hypothyroidism was associated with an increased risk of cognitive impairment, particularly MCI (8). However, most previous studies have focused on overt or subclinical thyroid dysfunction, whereas the association between physiological variations in thyroid hormones within the reference range and cognitive performance remains insufficiently clarified (9).

Among circulating thyroid hormones, free triiodothyronine (FT3) represents the principal biologically active form and may be particularly relevant to cognitive function (5). Beyond its direct neurobiological effects, FT3 is closely involved in glucose and lipid metabolism, mitochondrial regulation, and systemic energy homeostasis (10, 11). Experimental studies further suggest that thyroid hormone supplementation may improve hippocampal insulin signaling, neuroinflammation, and memory-related impairment in animal models (12, 13).

Studies have suggested an association between thyroid dysfunction, including subclinical hypothyroidism, and insulin resistance, although existing findings remain inconsistent (14). Thyroid hormones may influence insulin signaling and glucose metabolism through their regulatory effects on systemic energy homeostasis and metabolic pathways (15). Abnormal thyroid hormone signaling may therefore interact with insulin resistance, while altered metabolic status may also affect thyroid hormone activation, bioavailability, and tissue-level action (16, 17). The triglyceride-glucose (TyG) index, calculated from fasting triglyceride and fasting blood glucose levels, is a simple and practical surrogate marker of insulin resistance (18). Increasing evidence has associated the TyG index with T2DM, cerebrovascular disease, cerebral small vessel disease, and cognitive dysfunction (19). Nevertheless, the relationship between the TyG index and MCI in patients with T2DM remains insufficiently characterized, especially among individuals with normal thyroid function.

Moreover, whether FT3 and the TyG index are independently or jointly associated with MCI in euthyroid patients with T2DM, and whether these associations are nonlinear, remains unclear. Therefore, this study aimed to investigate the individual and combined associations of FT3 and the TyG index with MCI in euthyroid patients with T2DM, and to evaluate their potential value for cognitive risk stratification in this specific population.

## 2 Methods

### 2.1 Subjects

**Figure 1:**
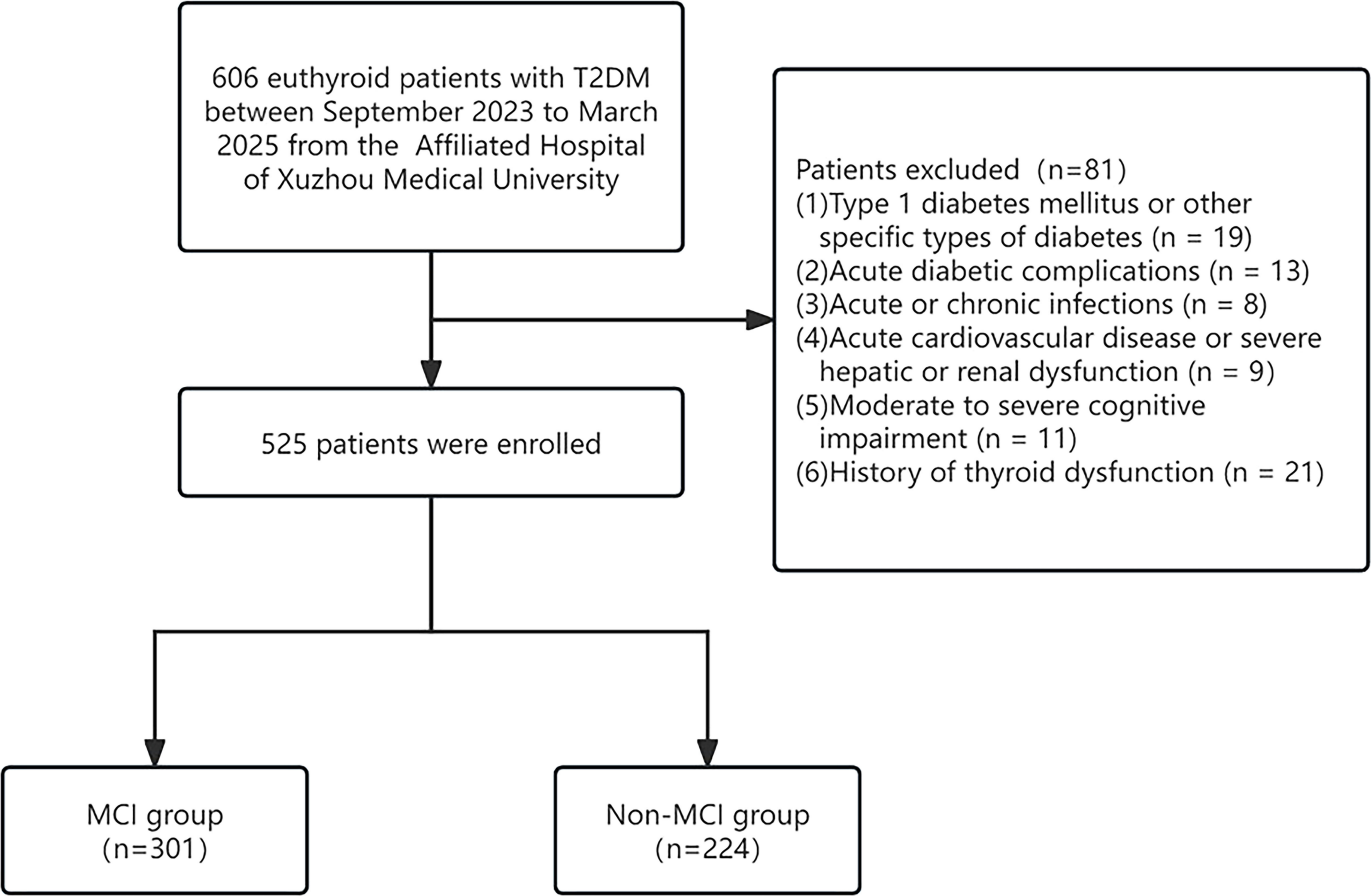
Flow chart of the study population.

This retrospective cross-sectional study included a total of 525 euthyroid patients with T2DM who were hospitalized at the Affiliated Hospital of Xuzhou Medical University between September 2023 and March 2025. All participants underwent routine biochemical examinations, thyroid function assessments, and neuropsychological testing. The study flowchart is presented in **Figure 1**. All recruited patients with T2DM met the diagnostic criteria of the American Diabetes Association (ADA) (20).

#### 2.1.1 Inclusion criteria

1. Age between 40 and 75 years;
2. Euthyroid status, defined by thyroid function tests within the following reference ranges: FT3, 3.10–6.80 pmol/L; FT4, 12.0–22.0 pmol/L; and TSH, 0.27–4.20 mIU/L. Normal thresholds for thyroid-related antibodies were defined as TPOAb 0.00–30.00 IU/mL and TGAb 0.00–95.00 IU/mL;
3. Diagnosis of T2DM according to the ADA criteria (20);
4. Normal cognitive function or MCI, defined by an education-adjusted Montreal Cognitive Assessment (MoCA) score ≥19. One additional point was added for participants with ≤12 years of formal education in accordance with standard MoCA scoring procedures.

#### 2.1.2 Exclusion criteria

1. Presence of acute diabetic complications, including diabetic ketoacidosis (DKA) and hyperosmolar hyperglycemic state (HHS), or treatment-related complications such as lactic acidosis (LA) and hypoglycemic coma (HC);
2. Moderate to severe cognitive impairment, defined as a MoCA score <19;
3. Current use of medications known to affect cognitive function, such as antipsychotics or sedative agents;
4. History of severe psychiatric disorders, neurological diseases, or head trauma or neurosurgical procedures;
5. History of thyroid dysfunction or thyroid surgery;
6. Acute or chronic infection, acute cardiovascular or cerebrovascular disease, severe hepatic or renal dysfunction, pregnancy, or malignancy.

This retrospective cross-sectional study was conducted in accordance with the Declaration of Helsinki and was approved by the Institutional Review Board (IRB) of the Affiliated Hospital of Xuzhou Medical University (approval number: XYFY2023-KL030-01). Due to the retrospective nature of the study, the requirement for informed consent was waived.

### 2.2 Data collection

Demographic and clinical data, including age, sex, educational level, duration of diabetes, and hypertension status, were collected. Height, weight, and blood pressure were measured in a quiet environment using standardized procedures. Hypertension (HPT) was operationally defined as a documented history of physician-diagnosed hypertension, current oral antihypertensive treatment, or admission systolic blood pressure ≥140 mmHg and/or diastolic blood pressure ≥90 mmHg, according to current hypertension guideline thresholds (21). Body mass index (BMI) was calculated as weight divided by height squared (kg/m^2^). All participants were required to fast overnight for at least 8 hours before blood sample collection. Biochemical measurements included glycated hemoglobin (HbA1c), fasting blood glucose (FBG), total cholesterol (TC), low-density lipoprotein cholesterol (LDL-C), high-density lipoprotein cholesterol (HDL-C), triglycerides (TG), and uric acid (UA). All laboratory analyses were performed using certified fully automated biochemical analyzers. The TyG index was calculated using the following formula:

TyG index = ln [fasting TG (mg/dL) × FBG (mg/dL)/2]. Thyroid function was assessed using a UniCel Dxi 800 Access Immunoassay System (Beckman Coulter, USA) and measured by the microparticle enzyme immunoassay (MEIA) method.

#### 2.2.1 Assessment of cognitive function and MoCA-based classification

Cognitive function was evaluated after admission using the Montreal Cognitive Assessment (MoCA), a 30-point cognitive screening instrument. Assessments were conducted by two uniformly trained physicians, and results were recorded. The MoCA has been shown to be a sensitive tool for detecting MCI, and previous studies in Chinese middle-aged and older adults have supported its utility for MCI screening (22). In accordance with standard MoCA scoring procedures, one additional point was added for participants with ≤12 years of formal education. In this study, cognitive status was classified using a MoCA-based operational definition: adjusted MoCA scores ≥26 indicated normal cognition, whereas scores of 19–25 were categorized as MoCA-defined MCI. Participants with adjusted MoCA scores <19 were excluded to minimize the inclusion of individuals with moderate-to-severe cognitive impairment (23). Based on these criteria, participants were categorized into the MCI group (n = 301) and the non-MCI group (n = 224).

### 2.3 Statistical analysis

Statistical analyses were performed using SPSS version 26.0 and R software version 4.3.1. Continuous variables were tested for normality and are presented as mean ± standard deviation or median (interquartile range), as appropriate. Group comparisons were conducted using independent-samples *t* tests or Mann–Whitney U tests. Categorical variables are expressed as number (percentage) and were compared using the chi-square test.

Logistic regression analyses were used to evaluate the associations of FT3 and the TyG index with cognitive impairment, with odds ratios (ORs) and 95% confidence intervals (CIs) calculated. Potential confounding factors were adjusted in stepwise multivariable models. Restricted cubic spline (RCS) analyses were applied to explore dose–response relationships and to assess potential nonlinear associations. Stratified analyses were performed across predefined subgroups, and interaction terms were included to evaluate the robustness of the associations.

Receiver operating characteristic (ROC) curve analyses were used to assess the discriminative performance of FT3, the TyG index, and their combined model. The area under the curve (AUC) was calculated, and differences between models were compared using the DeLong test. All statistical tests were two-sided, and *P* < 0.05 was considered statistically significant.

Calibration analysis was performed to evaluate the agreement between predicted and observed probabilities of cognitive impairment for the combined FT3 - TyG model. Decision curve analysis (DCA) was further conducted to assess the potential clinical net benefit of the model across different threshold probabilities.

## 3 Result

### 3.1 Baseline characteristics

Baseline characteristics of the study population are summarized in **Table 1**. A total of 525 diabetic patients with normal thyroid function were included, comprising 224 without cognitive impairment (Non-MCI, 42.7%) and 301 with MCI (57.3%). Compared with the non-MCI group, patients with MCI were older, had a longer disease duration, lower BMI, UA and FT3 levels, higher FBG, higher HbA1c, higher TyG index, and a higher prevalence of hypertension (all *P*<0.05). In addition, patients with MCI had a relatively low level of education (*P*<0.05). No significant intergroup differences were observed in sex, TG, TC, LDL-C, HDL-C or thyroid antibodies (all *P*>0.05). Although FT4 and TSH levels differed between groups, all values remained within the normal reference ranges, consistent with euthyroid status in the study population.

**Table 1:** Baseline characteristics.

Variables	Total (n = 525)	Non-MCI (n = 224)	MCI (n = 301)	t/z/χ2	*P*	Effect size (d/r/Cramér’s V)
Age, years	55.07 ± 10.17	49.66 ± 8.69	59.09 ± 9.30	-11.83	<0.001	-1.04
BMI, kg/m^2^	25.46 ± 4.17	26.26 ± 4.76	24.87 ± 3.57	3.65	<0.001	0.33
Sex, n(%)				1.53	0.216	0.05
female	194 (36.95)	76 (33.93)	118 (39.20)			
male	331 (63.05)	148 (66.07)	183 (60.80)			
Education, n(%)				52.99	<0.001	0.32
college degree and above	178 (33.90)	115 (51.34)	63 (20.93)			
high school and below	347 (66.10)	109 (48.66)	238 (79.07)			
HPT, n(%)	173 (32.95)	54 (24.11)	119 (39.53)	13.84	<0.001	0.16
Duration of diabetes, year	6.00 (1.00, 10.00)	4.00 (1.00, 10.00)	8.00 (3.00, 14.00)	-5.67	<0.001	-0.25
UA, umol/L	292.00 (240.00, 356.00)	312.50 (246.75, 374.25)	279.00 (231.00, 337.00)	-3.96	<0.001	-0.17
FBG, mmol/L	7.94 (6.49, 10.37)	7.37 (5.68, 9.45)	8.54 (7.06, 10.95)	-5.17	<0.001	-0.15
HbA1c, %	8.00 (6.83, 9.66)	7.50 (6.60, 9.20)	8.51 (7.04, 9.87)	-3.85	<0.001	-0.17
TC, mmol/L	4.69 (4.07, 5.40)	4.79 (4.13, 5.40)	4.62 (4.01, 5.40)	-1.24	0.215	-0.05
TG, mmol/L	1.62 (1.12, 2.56)	1.60 (1.02, 2.49)	1.66 (1.14, 2.59)	-1.02	0.308	-0.04
HDL-C, mmol/L	0.94 (0.80, 1.12)	0.96 (0.82, 1.13)	0.92 (0.79, 1.11)	-1.68	0.093	-0.07
LDL-C, mmol/L	2.47 (1.95, 3.06)	2.56 (1.96, 3.16)	2.42 (1.92, 3.03)	-0.69	0.488	-0.03
FT3, pmol/L	4.52 (4.04, 5.10)	4.99 (4.44, 5.54)	4.20 (3.81, 4.71)	-10.25	<0.001	-0.45
FT4, pmol/L	17.60 (16.00, 19.30)	17.10 (15.60, 18.50)	18.27 (16.48, 19.74)	-4.75	<0.001	-0.21
TSH, mIU/L	2.06 (1.37, 2.89)	1.82 (1.27, 2.46)	2.28 (1.49, 3.13)	-4.79	<0.001	-0.21
A-TG, IU/L	7.73 (4.92, 17.00)	7.59 (4.85, 14.83)	7.87 (4.98, 20.50)	-1.25	0.210	-0.05
A-TPO, IU/L	2.01 (1.10, 4.61)	1.93 (1.10, 4.56)	2.08 (1.14, 4.70)	-0.13	0.894	-0.01
TyG	9.24 (8.83, 9.83)	9.16 (8.55, 9.71)	9.29 (8.92, 9.87)	-2.80	0.005	-0.12

Data are presented as mean ± SD, median (Q1, Q3), or n (%), as appropriate. Effect sizes were reported as Cohen’s d for normally distributed continuous variables, r for non-normally distributed continuous variables, and Cramér’s V for categorical variables. For continuous variables, effect sizes were calculated as the standardized difference between the Non-MCI and MCI groups. Abbreviations: MCI, mild cognitive impairment; BMI, body mass index; HPT, hypertension; UA, uric acid; FBG, fasting blood glucose; HbA1c, hemoglobin A1c; TC, total cholesterol; TG, triglycerides; HDL-C, high-density lipoprotein cholesterol; LDL-C, low-density lipoprotein cholesterol; FT3, free triiodothyronine; FT4, free thyroxine; TSH, thyroid-stimulating hormone; A-TG, anti-thyroglobulin antibody; A-TPO, anti-thyroid peroxidase antibody; TyG, triglyceride-glucose.

### 3.2 Univariate logistic regression analysis of cognitive impairment

Univariate logistic regression analysis (**Table 2**) showed that age, education, HPT, duration of diabetes, BMI, UA, HbA1c, the TyG index, and FT3 were significantly associated with cognitive impairment (all *P*<0.05). Specifically, older age, presence of HPT, longer duration of diabetes, higher HbA1c, and higher TyG index were associated with higher odds of cognitive impairment. In contrast, higher education level, higher BMI, higher UA, and higher FT3 levels were associated with lower odds of cognitive impairment. Sex was not significantly associated with cognitive impairment [OR = 0.80, 95% CI 0.56–1.14, *P* = 0.216].

**Table 2:** Univariate logistic regression analysis.

Variables	β	S.E	Z	*P*	OR (95%CI)
Age	0.11	0.01	9.68	<0.001	1.12 (1.09 ~ 1.14)
Sex (male)	-0.23	0.18	-1.24	0.216	0.80 (0.56 ~ 1.14)
BMI	-0.08	0.02	-3.66	<0.001	0.92 (0.88 ~ 0.96)
Education					
college degree and above					1.00 (Reference)
high school and below	1.38	0.19	7.10	<0.001	3.99 (2.72 ~ 5.84)
HPT	0.72	0.20	3.69	<0.001	2.06 (1.40 ~ 3.02)
Duration of diabetes	0.08	0.02	5.42	<0.001	1.09 (1.05 ~ 1.12)
UA	-0.01	0.00	-4.04	<0.001	0.99 (0.99 ~ 0.99)
HbA1c	0.15	0.04	3.42	<0.001	1.16 (1.07 ~ 1.27)
TyG	0.30	0.11	2.74	0.006	1.34 (1.09 ~ 1.66)
FT3	-1.44	0.15	-9.43	<0.001	0.24 (0.18 ~ 0.32)

BMI, body mass index; UA, uric acid; HPT, hypertension; FT3, Free triiodothyronine; HbA1c, hemoglobin A1c; TyG, triglyceride-glucose; OR, odds Ratio; CI, confidence Interval.

### 3.3 Multivariable logistic regression analysis of cognitive impairment

Three multivariable logistic regression models were constructed to explore the independent associations of FT3 and the TyG index with cognitive impairment. Model 1 included age and sex. Model 2 included age, sex, education level, HPT, duration of diabetes, BMI, UA and HbA1c. Model 3 further included FT3 and the TyG index in addition to all covariates included in Model 2. As shown in **Table 3**, age was significantly associated with cognitive impairment in Model 1. After further adjustment in Model 2, age and education level remained independently associated with cognitive impairment. In the fully adjusted model (Model 3), both FT3 and the TyG index were independently associated with cognitive impairment. Specifically, higher FT3 levels were associated with lower odds of cognitive impairment, whereas a higher TyG index was associated with higher odds of cognitive impairment.

**Table 3:** Multivariable logistic regression models for mild cognitive impairment.

Variables	Model 1 OR (95% CI)	*P*	Model 2 OR (95% CI)	*P*	Model 3 OR (95% CI)	*P*
Age (per year)	1.12 (1.09~1.14)	<0.001	1.09 (1.06~1.12)	<0.001	1.09 (1.06~1.12)	<0.001
Sex (male)	0.99 (0.66~1.49)	0.964	1.29 (0.81~2.05)	0.280	1.71 (1.03~2.83)	0.038
High school and below	—		2.68 (1.72~4.16)	<0.001	2.16 (1.34~3.48)	0.002
HPT	—		1.76 (1.11~2.79)	0.016	1.42 (0.86~2.35)	0.172
Duration of diabetes	—		1.03 (0.99~1.06)	0.155	1.03 (0.99~1.07)	0.173
BMI	—		0.98 (0.92 ~ 1.04)	0.455	1.00 (0.93~1.07)	0.989
UA	—		1.00 (1.00~1.00)	0.381	0.99 (0.99~0.99)	0.029
HbA1c	—		1.15 (1.04~1.28)	0.005	1.00 (0.89~1.12)	0.957
TyG	—		—		1.94 (1.42~2.66)	<0.001
FT3	—		—		0.33 (0.24~0.48)	<0.001

BMI, body mass index; UA, uric acid; HPT, hypertension; FT3, free triiodothyronine; HbA1c, hemoglobin A1c; TyG, triglyceride-glucose index; OR, odds ratio; CI, confidence interval.

“—” indicates that the variable was not included in the corresponding model.

Model 1 included age and sex.

Model 2 included age, sex, education level, HPT, duration of diabetes, BMI, UA, and HbA1c.

Model 3 included age, sex, education level, HPT, duration of diabetes, BMI, UA, HbA1c, FT3, and the TyG index.

### 3.4 Association patterns between FT3, TyG index and cognitive impairment

**Figure 2:**
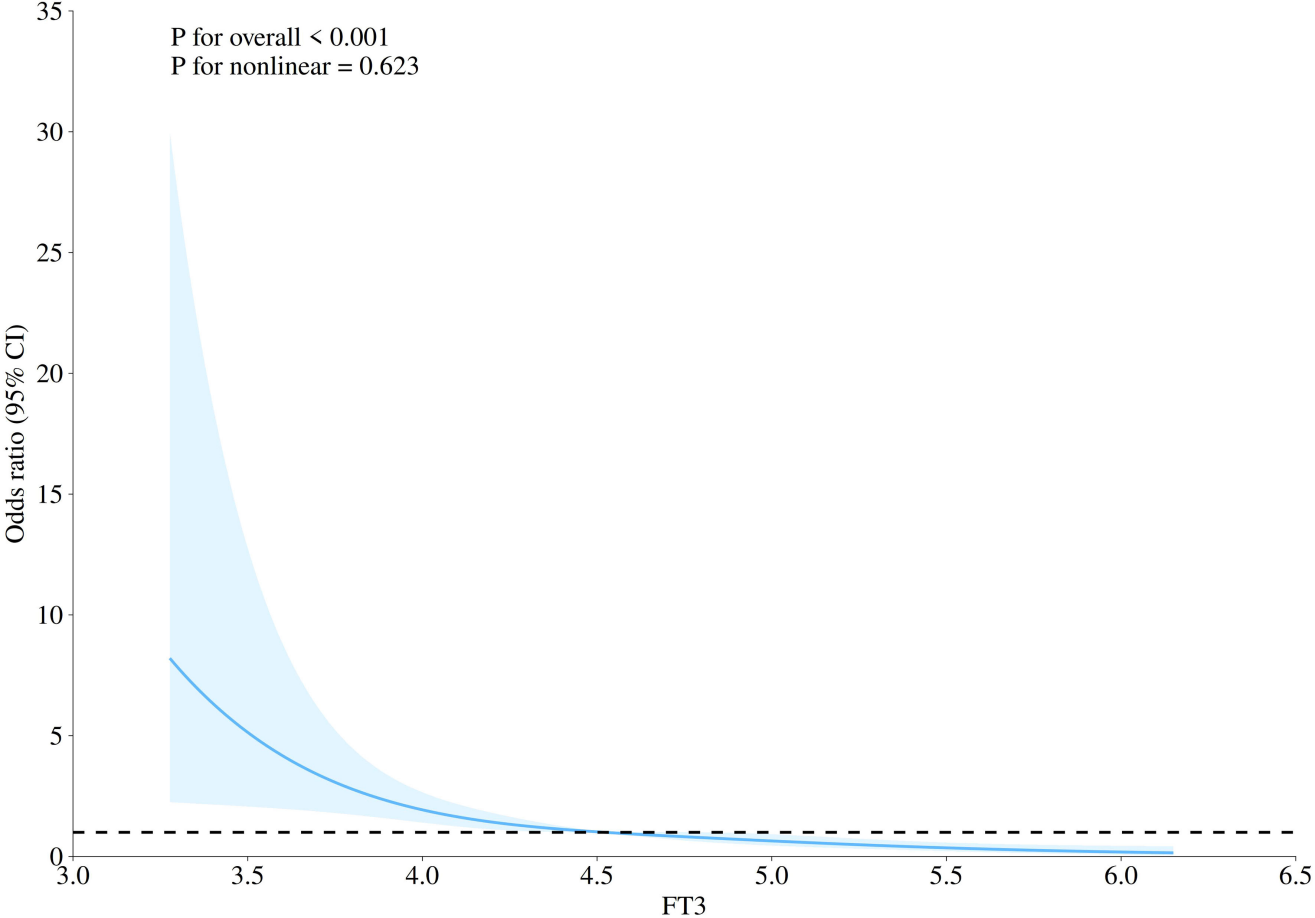
Restricted cubic spline curve of the association between FT3 and the odds of MCI. Adjusted for age, sex, education, BMI, HPT, HbA1c, UA, and duration of diabetes.

**Figure 3:**
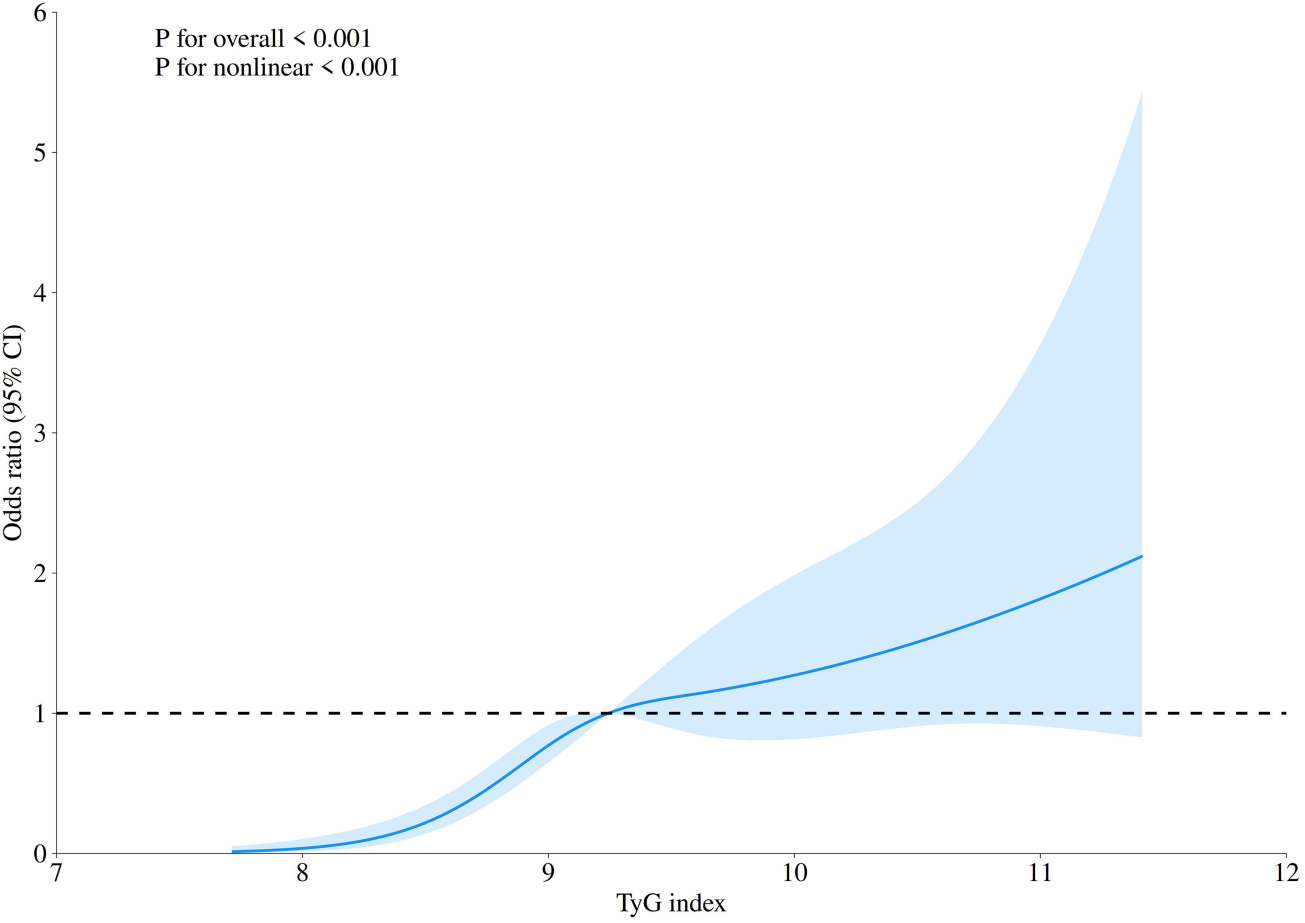
Restricted cubic spline curve of the association between the TyG index and the odds of MCI. Adjusted for age, sex, education, BMI, HPT, HbA1c, UA, and duration of diabetes.

Restricted cubic spline (RCS) analysis was employed to explore the association patterns between FT3, the TyG index and MCI, with results illustrated in **Figures 2**, **3**. Regarding FT3, the overall association with MCI was statistically significant (*P*<0.001), and the nonlinear test was not significant (*P* = 0.623), indicating a linear inverse association between FT3 levels and the odds of MCI. In contrast, for the TyG index, both the overall association with MCI (*P*<0.001) and the nonlinear test (*P*<0.001) were statistically significant, presenting a non-linear inverted asymmetric J-shaped relationship with an inflection point at TyG=9.24; specifically, when the TyG index was below the inflection point, the estimated odds of MCI increased slowly with higher TyG levels, whereas the estimated odds increased more rapidly when the TyG index exceeded the inflection point.

### 3.5 Subgroup analysis of the association between FT3 and cognitive impairment

**Figure 4:**
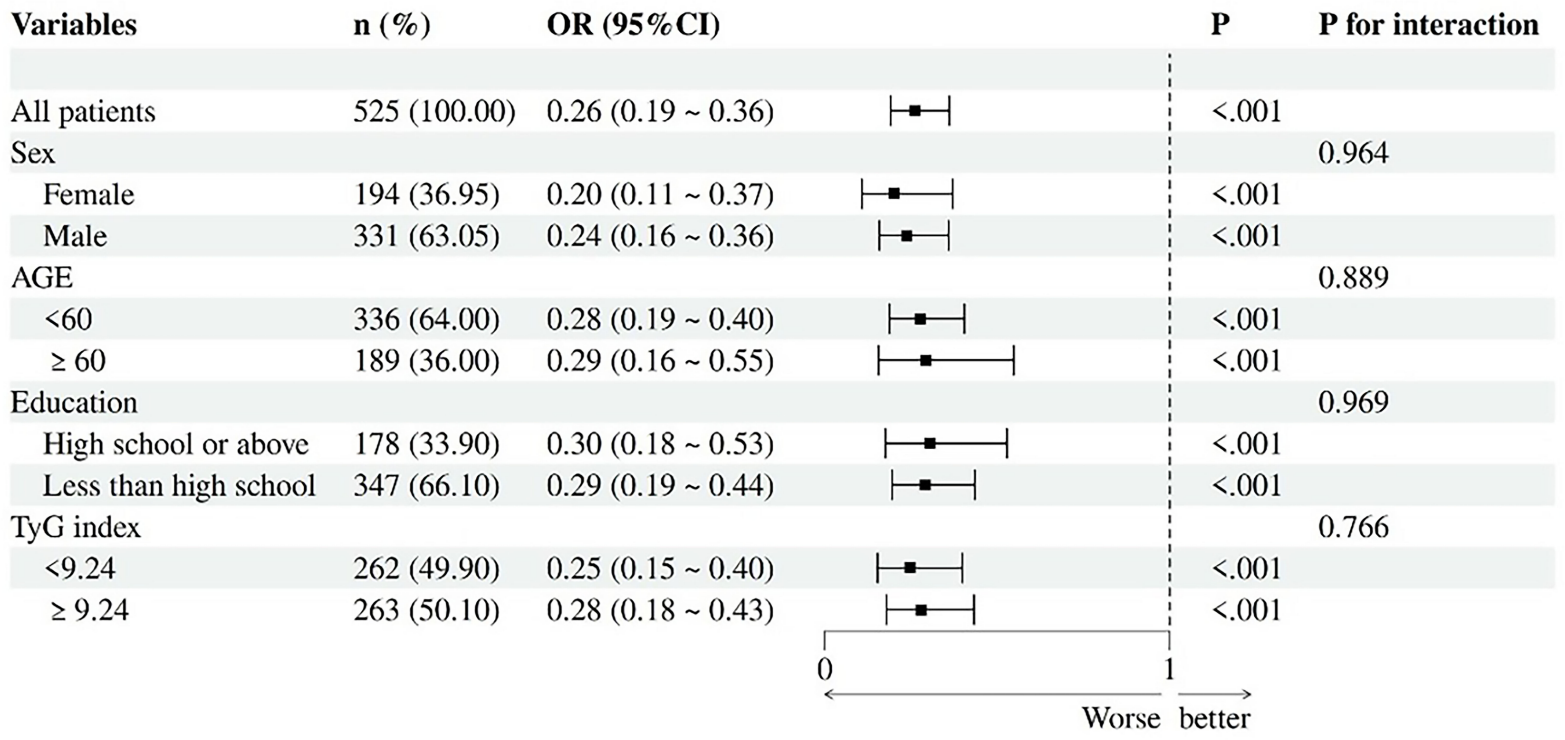
Subgroup analysis of the association between FT3 and cognitive impairment. All models were adjusted for BMI, HPT, HbA1c, UA, and duration of diabetes, except for the stratification variable.

Subgroup analysis was performed to evaluate the consistency of the inverse association between FT3 and cognitive impairment across different populations (**Figure 4**). The results showed that the inverse association of FT3 with cognitive impairment was consistent across subgroups stratified by age, sex, educational level, hypertension status, BMI, and the TyG index (all interaction *P*>0.05). No significant heterogeneity was observed, indicating that the association between FT3 and cognitive impairment was stable across these stratification factors.

### 3.6 Discriminative performance, calibration, and risk stratification value of FT3 and the TyG index

**Figure 5:**
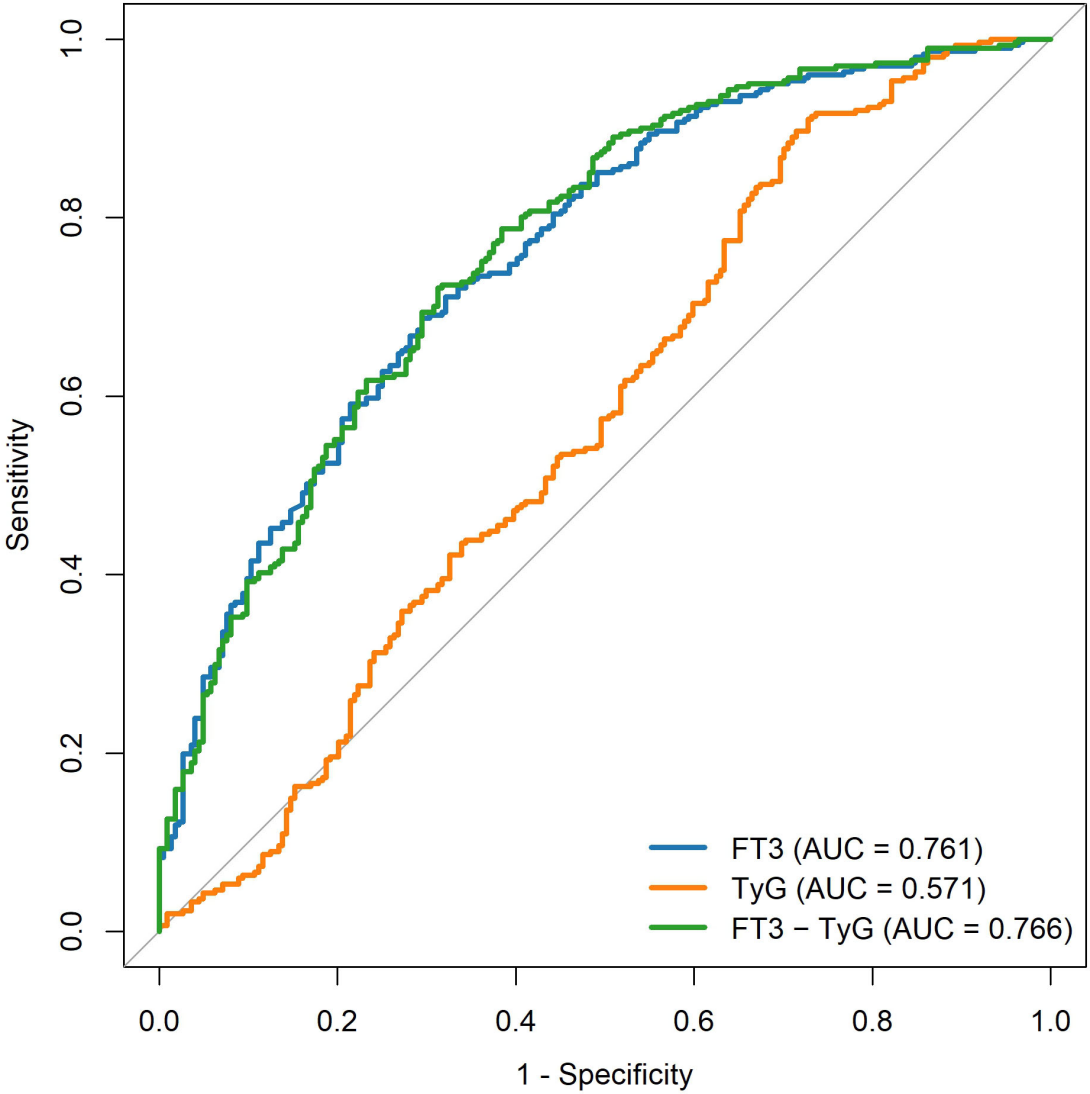
Receiver operating characteristic (ROC) curves evaluating the biomarker-based discriminative performance of FT3, the TyG index, and their combination for mild cognitive impairment.

**Figure 6:**
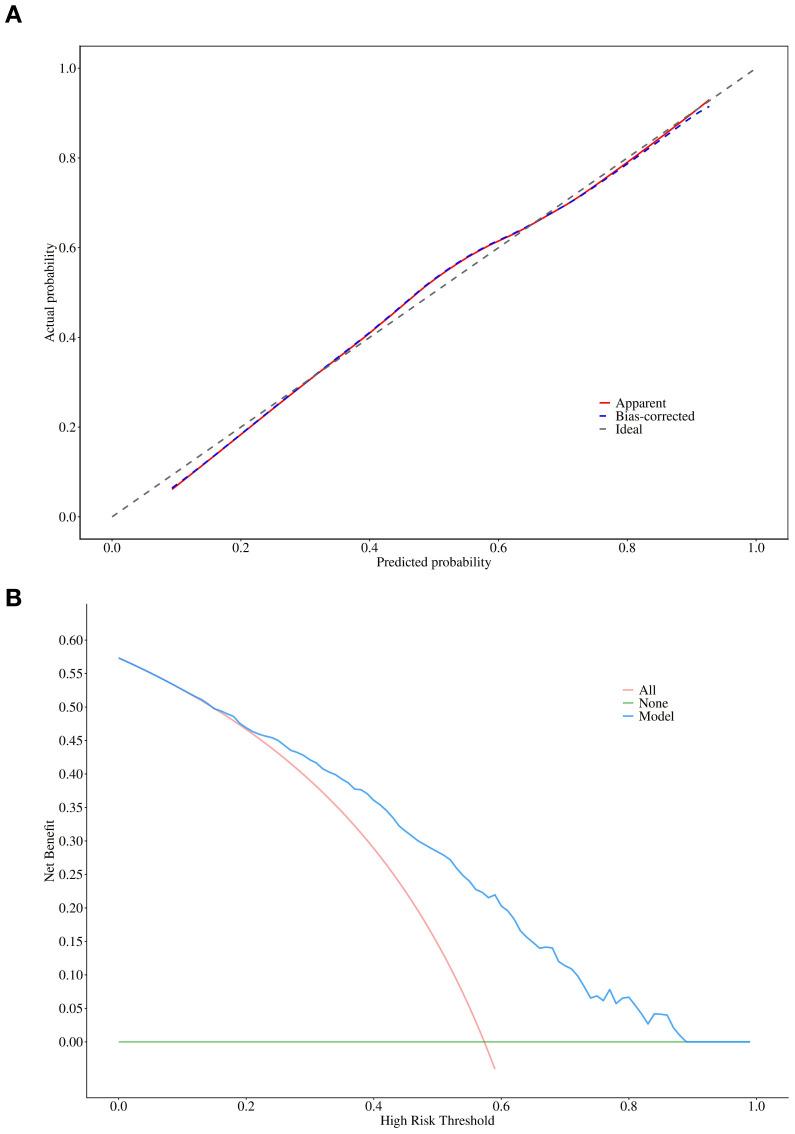
Calibration curve and decision curve analysis of the combined FT3 - TyG model. **(A)** Calibration curve. **(B)** Decision curve analysis.

The discriminative performance of FT3, the TyG index, and their combination for cognitive impairment was evaluated using receiver operating characteristic (ROC) curve analysis (**Figure 5**; **Table 4**). FT3 alone showed good discriminative ability, with an AUC of 0.761 (95% CI: 0.721–0.802), whereas the TyG index alone showed limited discrimination, with an AUC of 0.571 (95% CI: 0.520–0.622). The combined FT3 - TyG model yielded an AUC of 0.766 (95% CI: 0.726–0.807), representing only a small increase compared with FT3 alone. DeLong test analysis showed that this improvement was not statistically significant (Z = 0.786, *P* = 0.4319), indicating that adding TyG did not meaningfully enhance overall discriminative performance. Calibration analysis and decision curve analysis were further performed for the combined FT3 - TyG model (**Figure 6**). The calibration curve showed acceptable agreement between predicted and observed probabilities (**Figure 6A**), and decision curve analysis suggested potential clinical net benefit across a range of threshold probabilities (**Figure 6B**). Although TyG did not significantly improve overall discrimination beyond FT3, its significant nonlinear association with cognitive impairment and identified inflection point support its value in metabolic stratification.

**Table 4:** Comparison of ROC curves using DeLong’s test.

Comparison combination	AUC1 (95% CI)	AUC2 (95% CI)	Z value	*P*	95% CI for AUC difference
Combined Model vs. FT3 Alone	0.766 (0.726-0.807)	0.761 (0.721-0.802)	0.786	0.4319	-0.0074~0.0173
Combined Model vs. TyG Alone	0.766 (0.726-0.807)	0.571 (0.520-0.622)	6.643	<0.01	0.1374~0.2525

AUC1, Area under the curve of the first ROC curve (FT3-TyG combined prediction model for both comparisons); AUC2, Area under the curve of the second ROC curve (FT3 alone model, TyG alone model respectively).

## 4 Discussion

To our knowledge, this study is the first to demonstrate that lower FT3 levels within the euthyroid range are independently associated with MCI in patients with T2DM. Furthermore, we identified a nonlinear, asymmetric inverted J-shaped association between the TyG index and MCI risk, with an inflection point at TyG = 9.24. We also evaluated the biomarker-based discriminative performance of FT3, the TyG index, and their combination, and further explored the complementary role of TyG in metabolic stratification.

Previous studies have shown that lower FT3 levels are independently associated with an increased risk of cognitive impairment in older euthyroid adults (9). Zhang et al. further demonstrated that lower FT3 levels were associated with impairments in executive function and scene memory in patients with T2DM without diagnosed thyroid diseases (24). Based on these findings, our study provides new insights by showing that lower FT3 levels within the euthyroid range remained independently associated with MCI in patients with T2DM after multivariable adjustment. This suggests that subtle variations in biologically active thyroid hormone may be clinically relevant to cognitive vulnerability, even when thyroid function is conventionally classified as normal. FT3 is the biologically active form of thyroid hormone and may influence cognitive function through its involvement in neuronal differentiation, synaptogenesis, and cerebral energy metabolism (25, 26). In patients with T2DM, chronic hyperglycemia may interfere with deiodinase activity, peripheral conversion from FT4 to FT3, and tissue-level thyroid hormone sensitivity (27). Therefore, serum thyroid hormone concentrations within the reference range may not fully reflect thyroid hormone action at the tissue level. These literature-supported mechanisms may provide a possible explanation for the observed association between lower FT3 levels and MCI in the present euthyroid T2DM population.

In addition, our study clarified the nonlinear association between the TyG index and MCI in euthyroid patients with T2DM. Restricted cubic spline analysis showed an inflection point at TyG = 9.24, beyond which the estimated odds of prevalent MCI increased more steeply. This finding further extends previous studies that mainly reported an association between the TyG index and cognitive impairment (28). Our results suggest that, in patients with T2DM, cognitive vulnerability may become more evident once insulin resistance-related metabolic burden exceeds a certain threshold. An elevated TyG index reflects combined dysregulation of glucose and lipid metabolism (29). Persistent metabolic disturbance may adversely affect cognitive function through impaired brain insulin signaling, oxidative stress, blood–brain barrier disruption, and related neurovascular mechanisms (30, 31). Above mechanisms may partly explain why higher TyG levels, particularly those above the identified threshold, were associated with an increased probability of MCI in the present study.

The TyG index is calculated from fasting triglycerides and fasting glucose, both of which are routinely available and low-cost laboratory indicators in patients with T2DM (18). DeLong analysis showed that adding TyG to FT3 did not significantly improve the overall discriminative performance. Nevertheless, TyG may still have practical value as a complementary metabolic risk stratification marker. When considered together with its nonlinear association and the identified turning point, the TyG index may help identify patients with a heavier metabolic burden who may require closer cognitive monitoring and individualized metabolic management.

Older age is an important factor contributing to the decline in cognitive ability (32), longer diabetes duration is associated with a higher risk of cognitive impairment (33), and higher educational attainment may reduce the incidence of dementia, possibly through cognitive reserve mechanisms (34). Consistent with previous findings, patients with T2DM-MCI in our study were older, had a longer duration of diabetes, and had fewer years of education than those without MCI. After multivariable adjustment, older age and lower educational attainment remained significantly associated with MCI, whereas the association between diabetes duration and MCI was attenuated. This attenuation may be partly explained by the close relationship between diabetes duration and insulin resistance, as well as other metabolic disturbances.

Moreover, we found that patients with cognitive decline had higher FBG and HbA1c levels but lower UA levels. Although FT3 is the major biologically active form of thyroid hormones, patients with MCI in the present T2DM cohort also showed higher FT4 and TSH levels than those without MCI. While FT4 and TSH may also play important roles in cognitive function, the present study primarily focused on evaluating the discriminative value of FT3 and the TyG index. Physical function is a well-established risk factor for cognitive impairment in middle-aged and older adults with T2DM (35). However, not including these indicators (e.g., SPPB, gait speed) in our considerations may have limited the comprehensiveness of our risk stratification. Future studies should integrate physical function measures with metabolic and endocrine markers to construct a more comprehensive cognitive risk assessment framework for patients with T2DM.

Several limitations of this study should be acknowledged. First, the cross-sectional design precludes causal inference. Second, this was a single-center study based on hospitalized patients, which may restrict the generalizability of the findings. Third, cognitive function was assessed using the MoCA, which is a screening tool rather than a diagnostic instrument. Therefore, misclassification bias may exist. Future studies using comprehensive neuropsychological testing are needed to strengthen cognitive classification. Furthermore, it is necessary to conduct multi-center studies, as well as external validation and relevant biomarker detection, in order to further confirm these findings.

## 5 Conclusion

In conclusion, lower FT3 levels within the euthyroid range were independently associated with MCI in patients with T2DM, suggesting that subtle changes in biologically active thyroid hormone may contribute to cognitive vulnerability even when thyroid function is conventionally normal. Moreover, the TyG index showed a nonlinear association with MCI, with a threshold at TyG = 9.24, beyond which the likelihood of MCI increased more markedly. These findings indicate that FT3 and the TyG index may provide complementary value for cognitive risk stratification in euthyroid patients with T2DM. Future longitudinal studies incorporating physical function measures are warranted to confirm these findings and improve comprehensive cognitive risk assessment in this population.

## Data Availability

The raw data supporting the conclusions of this article will be made available by the authors, without undue reservation.
